# Usefulness of Ultrasound in Assessing the Impact of Bariatric Surgery on Body Composition: a Pilot Study

**DOI:** 10.1007/s11695-023-06510-9

**Published:** 2023-02-27

**Authors:** A. Simó-Servat, M. Ibarra, M. Libran, C. Quirós, N. Puértolas, N. Alonso, V. Perea, R. Simó, MJ. Barahona

**Affiliations:** 1 Department of Endocrinology and Nutrition, Hospital Universitari MútuaTerrassa, Plaça del Doctor Robert, 5, 08221 Terrassa, Barcelona, Spain; 2 Department of Medicine, Autonomous University of Barcelona, Bellaterra, Barcelona, Spain; 3 Department of General Surgery, Hospital Universitari MútuaTerrassa, Terrassa, Barcelona, Spain; 4 Diabetes and Metabolism Research Unit, Vall d’Hebron Research Institute and CIBERDEM (ISCIII), Autonomous University of Barcelona, Terrassa, Barcelona, Spain

**Keywords:** Musculoskeletal ultrasound, Bariatric surgery, Sarcopenic obesity

## Abstract

**Background:**

Bariatric surgery (BS) has a significant impact on body composition. The purpose of the study is to evaluate the usefulness of musculoskeletal ultrasound (MUS) to bioelectrical impedance (BIA) in the follow-up of patients undergoing BS in terms of body composition and quality of life (QoL).

**Methods:**

This is a prospective pilot study including 32 subjects (75% female, mean age: 49.15 ± 1.9 years) who underwent BS. Fat mass (FM), lean mass (LM), and skeletal muscle index (SMI) were calculated by BIA. MUS measured subcutaneous fat (SF) and thigh muscle thickness (TMT) of the quadriceps. QoL was assessed by the Moorehead-Ardelt questionnaire. All these measurements were performed 1 month prior to BS and at 12-month follow-up.

**Results:**

The mean BMI decreased by 6.63 ± 1.25 kg/m^2^ (*p*=0.001). We observed significant reductions in FM (*p*=0.001) and SF (*p*=0.007) and in LM (*p*=0.001) but not in SMI and TMT. We found a correlation between the FM and SF (pre-surgical, *r*=0.42, *p*=0.01; post-surgical, *r*=0.52, *p*=0.003) and between SMI and TMT (pre-surgical, *r*=0.35, *p*=0.04; post-surgical, *r*=0.38, *p*=0.03). QoL test showed significant improvement (*p*=0.001). In addition, a correlation between the QoL questionnaire and TMT post-surgery (*r*=0.91, *p*=0.019) was observed. However, we did not find any statistically significant correlation between QoL assessment and SMI or LM.

**Conclusions:**

Our results suggest that MUS can be complementary to BIA for the evaluation and the follow-up of body composition after BS. TMT of quadriceps can provide relevant information about regional sarcopenia and has a significant correlation with QoL.

**Graphical Abstract:**

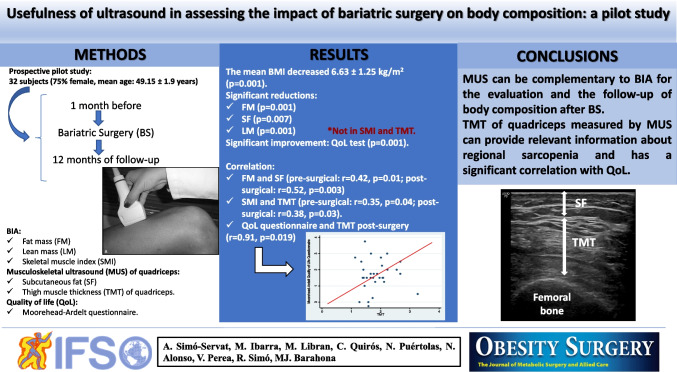

## Introduction

Bariatric surgery (BS) can achieve sustained significant weight loss providing major health benefits by reducing fat mass (FM) as it is an important determinant of co-morbidity [1, 2]. However, after BS, a decrease in muscle mass also occurs. This loss of muscle mass has been associated with lower psychological health and quality of life (QoL) and higher prevalence of type 2 diabetes, hypertension, and all-cause mortality [3, 4]. Accordingly, preservation of muscle mass during weight loss after BS is clinically relevant [5]. A recent study showed that after gastric sleeve surgery, patients lost an average of 10% lean mass (LM) in the first month and 17% loss after 1 year. However, the change in weight and the subsequent change in the BMI do not reflect the changes in FM and LM, the two main compartments of body composition [6]. Among the many relevant techniques for measuring muscle mass, muscle ultrasonography (MUS) is an emerging tool in clinical practice. MUS is a simple, real-time, noninvasive, radiation-free, low-cost, and easily transportable, as well as valid and reliable, method to estimate muscle mass. There are clinical trials exploring MUS assessment for the diagnosis of sarcopenia [7, 8], but there is a lack of research on the role of MUS in diagnosing sarcopenic obesity (SO) and in evaluating the changes in LM after BS [9]. MUS would allow an accessible and useful assessment of sarcopenia in patients with obesity after BS and could be used as an alternative or complementary method to the conventionally used skeletal muscle mass index (SMI) measured by BIA or DEXA [10]. Moreover, MUS can detect regional sarcopenia of a specific group of muscles as the quadriceps. This muscle group is essential to carry out basic functions for the autonomy of an individual since it is essential for walking [11] and consequently for QoL. On this basis, thigh muscle thickness (TMT) and subcutaneous fat (SF) measurements obtained by ultrasound (US) of quadriceps are new, easily accessible parameters that we can introduce in clinical practice to improve the study of body composition of our patients. In fact, morphological characteristics based on US measures of the quadriceps muscle could be used for the screening and initial evaluation of SO in candidates for BS and, especially, in their follow-up. This approach will allow us to gain new insights into the potential usefulness of MUS [12].

The aim of this longitudinal pilot study is to examine whether MUS has an added value to BIA in terms of monitoring the changes of body composition and QoL after BS.

## Material and Methods

We performed a prospective observational pilot study carried out in our hospital. Subjects were recruited from the outpatient Obesity Unit between February 2019 and November 2020. Subjects were candidates for BS when their BMI was higher than 35 kg/m^2^ with comorbidities or BMI> 40 kg/m^2^. The study follows the STROBE guidelines for prospective studies [13]. Exclusion criteria were age ≥65 years, pregnancy, and patients with clinical or personal characteristics that make monitoring difficult: drug or alcohol addiction, severe psychological, or psychiatric disorders. BIA and MUS tests as well as Moorehead-Ardelt Quality of Life Questionnaire were conducted during the same visit. All these assessments were carried out 1 month before surgery and at 12-month follow-up. The Hospital’s Ethics Committee approved all the procedures carried out in the study, and all subjects signed the informed consent before their inclusion in the study.

The skeletal muscle mass (SM) was measured using BIA. This method measures the body composition according to the differences in electric impedance among biological tissues using Janssen’s equation: SM (kg) = [(height − 2 (cm)/BIA resistance (ohms) × 0.401) + (gender (men = 1, women = 0) × 3.825) + (age (years) × −0.071)] + 0.5102. We used the SMI of Baumgartner to identify sarcopenia in people with obesity, which was calculated using SMI = SM kg/height^2^ [14]. The BodyStat^®^ 1500 MDD model was used as previously described [12].

**Fig. 1:**
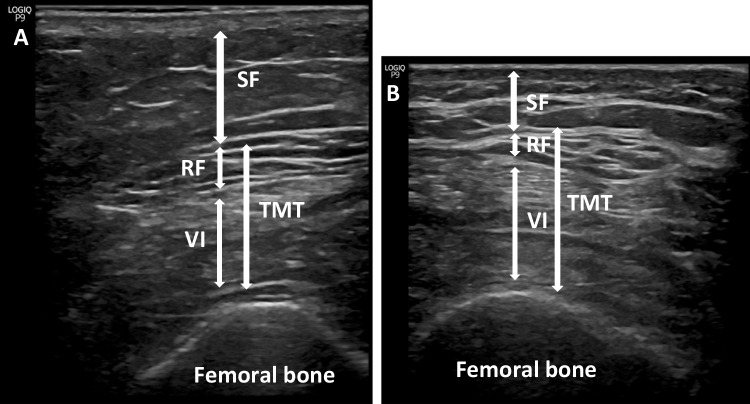
Measurement of subcutaneous tissue and thigh muscles using US. SF, subcutaneous fat; TMT, thigh muscle thickness; VI, vastus intermedius; RF, rectus femoris. **A** Representative image of participant (Id 17) before BS. **B** Representative image of participant (Id 17) after BS

US measurements were made with a sonographic US Logiq P9 (GE Healthcare) equipment muscle-skeleton B-model using a linear multifrequency transducer (4–11 Hz) with adequate use of contact gel and minimal pressure to avoid excessive compression of the muscle. Patient positioning was done in accordance with what is reported in the literature [12, 15–19] (Fig. 1). Sarcopenia mainly affects lower limbs, so the rectus femoris plus vastus intermedius were specifically selected to evaluate it [20], and its US evaluation was carried out according to the recommendations of the European Union Geriatric Medicine Society Sarcopenia Special Interest Group and in accordance with the previous literature [19]. A set of three consecutive measurements was performed, and the average value was reported as the TMT or SF. Data were reported in centimeters (cm) as means ± standard deviation. To avoid interindividual variability, the same physician (endocrinologist A. S-S) with 4 years of experience performed all measurements. To assess intra-observer reliability, we evaluated intraclass correlation coefficients (CVs) using 3 images taken on 3 separate days on 30 participants, and the results of CVs were 0.93 for TMT and 0.87 for SF.

### Statistical analysis

STATA statistical software version 14 (College Station, TX) was used. Continuous variables are expressed as mean ± standard deviation (SD) unless specified otherwise. Categorical variables are expressed with percentages. *T*-test was used to assess the differences between groups in continuous variables, Fisher test was used for categorical variables, and the Pearson’s correlation test was used to explore the relation between different variables. All analyses were 2-tailed, with *p* <0.05 considered statistically significant.

## Results

The baseline data of the participants are shown in Table 1. A total of 32 subjects were included: 24 female participants (75%), mean age of 49.15 ± 1.9 years. The mean baseline BMI was of 43.79 ± 4.95 kg/m^2^ and decreased 6.63 ± 1.25 kg/m^2^ (*p*=0.001).

**Table 1:** The baseline characteristics of the patients

Total sample size	32
Female	24 (75%)
Age (years) mean ± SD	49.15 ± 1.9
BMI (kg/m^2^) mean ± SD	43.79 ± 4.95
Gastric bypass Roux-en-Y (%)	26 (81.25)
Sleeve gastrectomy (%)	6 (18.74)
Diabetes Mellitus II (%)	10 (31.25)
Hypertension (%)	19 (59.4)
Dyslipidemia (%)	11 (34.4)

*BMI body mass index

**Fig. 2:**
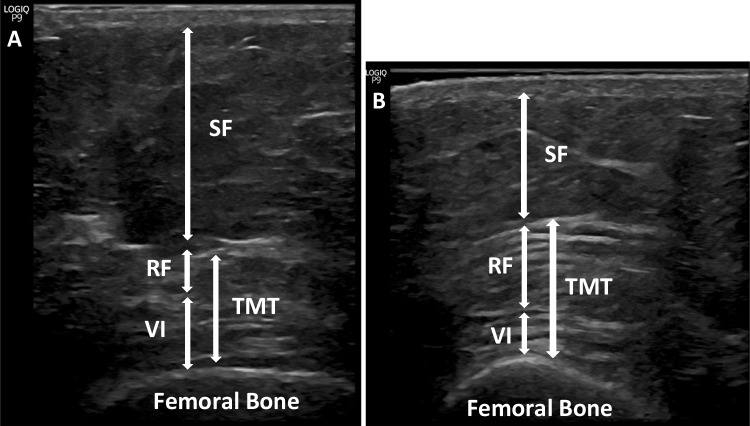
Measurement of subcutaneous tissue and thigh muscles using US. SF, subcutaneous fat; TMT, thigh muscle thickness; VI, vastus intermedius; RF, rectus femoris. **A** Representative image of participant (Id 25) before BS. **B** Representative image of participant (Id 25) after BS

The anthropometric parameters (FM and LM) assessed by BIA and the measurements performed using US (SF and TMT) are displayed in Table 2. We found significant reductions in FM (45.19 ± 1.34 vs 37.92 ± 1.81, *p*=0.001) and SF (1.27 ± 0.11 vs 1.03 ± 0.09, *p*=0.007). A significant reduction in LM (66.05 ± 2.42 vs 62.29 ± 2.42, *p*=0.001) was also observed, but not in SMI (*p*=0.057) and TMT (*p*=0.635). In fact, in 12 subjects, the TMT improved despite detecting a decrease in the LM. Specifically, the mean TMT of these 12 subjects increased (1.28 ± 0.13 vs 2 ± 0.16, *p*=0.0005). In Fig. 2, we can see the improvement of the TMT in a particular patient.

**Table 2:** Anthropometric parameters by BIA and US measurements pre- and post-BS

	Pre-surgery (mean ± SD)	Post-surgery (mean ± SD)	p*
LM (kg)	66.05 ± 2.42	62.29 ± 2.42	0.001
FM (%)	45.19 ± 1.34	37.92 ± 1.81	0.001
SMI (kg/m^2^)	10.19 ± 0.39	9.85 ± 0.39	0.057
TMT (cm)	1.74 ± 0.09	1.79 ± 0.1	0.635
SF (cm)	1.26 ± 0.1	1.06 ± 0.09	0.007

*SMI*, skeletal muscle mass index; *LM*, lean mass; *FM*, fat mass; *SF*, subcutaneous fat; *TMT*, thigh muscle thickness

*p <0.05 considered statistically significant

**Fig. 3:**
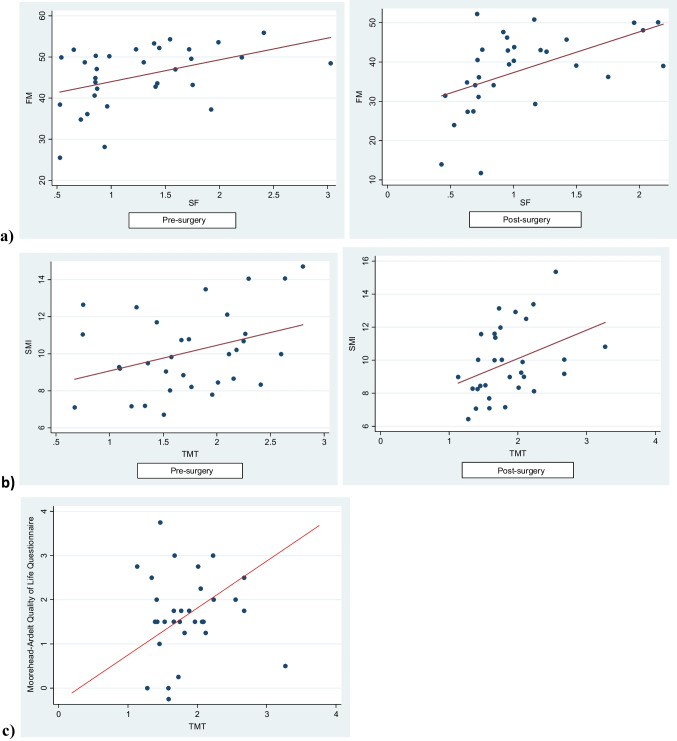
**a** A significant correlation was observed between fat mass (FM) by bioelectrical impedance analysis (BIA) and thigh muscle thickness (TMT) by ultrasound (US) (pre-surgical, *r*=0.42, *p*=0.01; post-surgical, *r*=0.52, *p*=0.003). **b** A significant correlation was observed between skeletal muscle mass index (SMI) assessed by BIA and TMT assessed by US (pre-surgical, *r*=0.35, *p*=0.04; post-surgical, *r*=0.38, *p*=0.03). **c** A significant correlation was observed between the Moorehead-Ardelt Quality of Life Questionnaire and TMT assessed by US post-surgery (*r*=0.91, *p*=0.0192).

We found a positive correlation between the FM and SF (pre-surgical, *r*=0.42, *p*=0.01; post-surgical, *r*=0.52, *p*=0.003) and between SMI and TMT (pre-surgical, *r*=0.35, *p*=0.04; post-surgical, *r*=0.38, *p*=0.03) (Fig. 3). A significant improvement in the Moorehead-Ardelt Quality of Life Questionnaire score (0.52 ± 0.27 vs 1.66 ± 0.17, *p*=0.001) was observed, and this increasing punctuation was better in the 12 subjects where the TMT rises (1.21 ± 0.21 vs 1.09 ± 0.31). Notably, we found a strong correlation between TMT assessed post-BS and the QoL test (*r*=0.91, *p*=0.0192) (Fig. 3). However, we did not find any statistically significant correlation between the QoL questionnaire and SMI or LM.

## Discussion

In the present study, changes in body composition were evaluated with BIA and MUS in patients undergoing BS at baseline and after 12-month follow-up. The results showed a loss of FM, LM, and SF but not of SMI and TMT. FM had the greatest relative and absolute change. In particular, the significantly higher change in FM and SF compared with LM and TMT before BS and after 12 months confirm a significant change in body composition that was already found in other studies [21]. We demonstrate a good correlation between pre- and post-surgery using US and BIA for assessing body composition. These good correlations have been observed between FM and SF, as well as between the SMI calculated by BIA and the TMT, thus supporting the usefulness of US to assess the follow-up of these patients.

In addition, US revealed that 12 patients had a significant improvement in TMT after surgery but not in LM and SMI. These 12 patients have a lower mean age (44 years) than the other 20 patients (52 years), with no differences regarding sex. They did not present a different LM and FM at baseline or greater weight reduction compared to the rest of the patients. So, this improvement in TMT could be due to increased physical activity after BS. Since MUS measures the muscle mass of the quadriceps, it provides relevant information about the specific group of muscles that the patient works out. By contrast, BIA does not show the same level of accuracy. Therefore, the patient could improve the QoL even if the total LM does not show improvement by BIA, since the improvement of the quadriceps can allow patient autonomy. In fact, we found a strong positive correlation between the QoL test and TMT post-BS. In addition, the improvement of QoL questionnaire punctuation (1.21 ± 0.21) in the 12 patients in whom TMT enhanced after BS was higher than in those patients (1.09 ± 0.31) without TMT increase. These findings suggest that MUS could be useful and complementary in determining regional sarcopenia, which directly affects their quality of life, opening another field of study about regional sarcopenia in determinate groups of muscles.

This prospective pilot study revealed that the changes in body composition produced by BS are also significant with US and that they correlate with BIA. The use of MUS to determine changes in body composition in SO population, and specifically in the candidates to for BS provides more insight into a correct evaluation of body composition after the intervention. Currently, in most centers, no body composition study is performed in a systematic or protocolized manner, probably due to a lack of resources. DEXA, CT, and MRI are methods that are not only difficult to access and expensive, but also have radioactive effects. In contrast, MUS is an increasingly accessible tool in the consultation that could guide us on body composition as a screening for SO. Since US can provide regional information of a muscle group, it could be a complementary technique to both the BIA and the dynamometer that provide functional information. Overall, our results point to further exploring the usefulness of US in this field and establishing cut-off points as a diagnostic method for regional sarcopenia.

The main limitation of our study is the small sample size and the lack of a control group of subjects with normal weight. Nevertheless, the study was aimed to evaluate the evolution after surgery and the patients represented their own control [22]. The disadvantages of US are principally the lack of standardization and its high dependence on the expertise and skills of the operator [23]. The interpretation of muscle–fat interfaces is limited due to similar acoustic impedance of muscle and fat tissues. Another drawback of the US technique is that the operator could cause measurement errors by applying the transducer to the skin with excessive pressure as this may compress the muscle [24]. In addition, we did not have data on muscle function. However, given that the quadriceps is an important muscle for mobility measurement, quadriceps thickness provides a useful surrogate of force [25–27]. The same physician performed all measurements, and this precluded the test reproducibility. Finally, this is a pilot study, and, therefore, larger studies are needed to confirm our results.

## Conclusions

Our results suggest that MUS can be complementary to BIA for the evaluation and the follow-up of body composition in patients operated on for BS. TMT of quadriceps rather than LM is strongly correlated with QoL, and, therefore, MUS is an easy, noninvasive, low-cost tool that provides relevant information about quadriceps sarcopenia, which is closely related to QoL.
